# The clinical relevance of outcomes used in late-onset Pompe disease: can we do better?

**DOI:** 10.1186/1750-1172-8-160

**Published:** 2013-10-12

**Authors:** Robin Lachmann, Benedikt Schoser

**Affiliations:** 1 National Hospital for Neurology and Neurosurgery, London, UK; 2 Friedrich-Baur Institut, Neurologische Klinik, Klinikum der Universität München, München, Germany

**Keywords:** Late-onset Pompe disease, Minimal clinically important difference, Outcome measures

## Abstract

Pompe disease/glycogen storage disease type II, is a rare, lysosomal storage disorder associated with progressive proximal myopathy, causing a gradual loss of muscular function and respiratory insufficiency. Studies of patients with late-onset Pompe disease have used endpoints such as the 6-minute walking test (6MWT) and forced vital capacity (FVC) to assess muscular and respiratory function during disease progression or treatment. However, the relevance of these markers to late-onset Pompe disease and the minimal clinically important difference (MCID) for these endpoints in late-onset Pompe disease have not yet been established. A literature search was carried out to identify studies reporting the MCID (absolute and relative) for the 6MWT and FVC in other diseases. The MCIDs determined in studies of chronic respiratory diseases were used to analyze the results of clinical studies of enzyme replacement therapy in late-onset Pompe disease. In 9 of the 10 late-onset Pompe disease studies reviewed, changes from baseline in the 6MWT were above or within the MCID established in respiratory diseases. Clinical improvement was perceived by patients in 6 of the 10 studies. In 6 of the 9 late-onset Pompe disease studies that reported FVC, the changes from baseline in percentage predicted FVC were above or within the MCID established in respiratory diseases and the difference was perceived as either an improvement or stabilization by patients. However, applying the 6MWT and FVC MCIDs from studies of chronic respiratory diseases to late-onset Pompe disease has several important limitations. Outcome measures in muscular dystrophies include composite measures of muscle function and gait, as well as Rasch-designed and validated tools to assess disease-related quality of life and activities of daily living. Given that the relevance to patients with late-onset Pompe disease of the 6MWT or FVC MCIDs established for chronic respiratory diseases is unclear, these measures should be evaluated specifically in late-onset Pompe disease and alternative outcome measures more specific to neuromuscular disease considered.

## Background

One of the key factors in the evaluation of an intervention in controlled clinical trials is the clinical relevance of the selected study endpoints or outcome measures, together with an understanding of what comprises a minimal clinically important difference (MCID) in these endpoints. Establishing the MCID for study endpoints allows the clinical relevance of efficacy data from published trial results to be determined. This is of particular relevance in studies that investigate treatment efficacy in chronic, progressive diseases such as the lysosomal storage disorders.

Late-onset Pompe disease (also known as glycogen storage disease type II or acid α-glucosidase deficiency) is a rare lysosomal storage disorder caused by a genetic deficiency in the enzyme acid α-glucosidase. Overall incidence ranges from 1 in 33,000 persons to 1 in 300,000 persons, depending on geographic region and ethnicity [1-3]. Late-onset Pompe disease may develop in children and adults of any age, and presents with a wide spectrum of clinical phenotypes [4-7]. Patients with late-onset Pompe disease usually present with progressive muscle weakness (often in a limb-girdle pattern) and loss of muscular function, leading to problems with activities of daily living (ADL), reduced mobility, and eventually wheelchair use. The disease also affects the respiratory muscles resulting in the need for ventilatory support in a high proportion of patients [6,8,9]. Patients with untreated late-onset Pompe disease have higher mortality rates compared with the general age- and gender-matched healthy population [8], while those treated with enzyme replacement therapy (ERT) experience a 59% reduction in mortality [10].

Long-term studies and clinical trials with ERT in late-onset Pompe disease commonly include exercise capacity (normally measured using the 6-minute walking test [6MWT]) and pulmonary function (forced vital capacity [FVC]) as outcome measures. However, the clinical relevance and impact on the patients’ ADL of any observed changes in the 6MWT distance (6MWD) and FVC (measured as percentage change in predicted FVC [% predicted FVC]) in studies of late-onset Pompe disease are currently unclear. Some information exists on the clinical relevance of these outcome measures for other chronic diseases, including respiratory diseases such as chronic obstructive pulmonary disease (COPD) and idiopathic pulmonary fibrosis (IPF), but the relevance and MCID of the 6MWT and FVC in late-onset Pompe disease have not been established and there is a need to look beyond the 6MWT and FVC as clinical endpoints.

In this article we determine the clinical relevance of 6MWD and % predicted FVC (main outcome measures), which are currently used to assess late-onset Pompe patients, to compare these with the parameters used in long-term studies in other neuromuscular disorders (NMDs) such as Duchenne/Becker muscular dystrophy (DMD/BMD), and to consider the potential clinical relevance of alternative clinical endpoints in late-onset Pompe disease.

## Methods

Three parallel literature searches were conducted using the PubMed database. The first search used the terms “6MWT or 6MWD”, “quality of life”, and “activities of daily living”. The second search used the terms “forced vital capacity”, “quality of life”, and “activities of daily living”. These two searches were not restricted to late-onset Pompe disease in order to identify studies in other diseases that could help interpret the clinical relevance of 6MWT and FVC as outcome measures. The final literature search was performed to identify endpoints commonly used in clinical trials in NMDs, and to investigate new endpoints and scoring systems currently under investigation in NMDs and other chronic diseases. The search was limited to articles from human studies, published in English, with full abstracts. The retrieved articles were scanned to identify papers which utilized a threshold of the 6MWD and FVC to indicate a change either in the patients’ ability to function or in the patients’ perception of their health. The reference lists of all relevant papers were also reviewed to identify any additional publications missed in the original literature search.

### Outcome measures used in late-onset Pompe disease

Studies of late-onset Pompe patients commonly report absolute or relative changes in the 6MWD and the change in FVC as outcome measures. Other measures used in long-term clinical trials involving late-onset Pompe patients include the Walton Gardner Medwin (WGM) score, maximum inspiratory pressure (MIP), maximum expiratory pressure (MEP), timed muscle function tests (e.g. modified Gowers’ maneuver), and quantitative muscle testing [11-15].

The 6MWT was originally developed as an integrated assessment of cardiac, pulmonary, circulatory, and muscular capacity in patients with moderate or severe lung disease, and provides a measure of the functional exercise level required to undertake daily physical activities [16]. The 6MWT allows patients to rest when needed and, therefore, provides a measure of submaximal exercise capacity, designed to reflect the physical effort used in ADL. In a study of healthy adults aged 40–80 years (median age 58 years), the mean baseline 6MWD was 571 m with significantly shorter distances observed in those aged ≥60 years [17]. In comparison, in patients with untreated late-onset Pompe disease a decline in walking ability is observed at a much younger age than in healthy subjects and, therefore, the normal age-related decline may occur from a lower base level of walking ability in most patients. Studies of late-onset Pompe patients have reported baseline 6MWDs from 246–340 m (Angelini et al. [12] mean age at study entry 43 years [range 7–72]; Regnery et al. [13] mean age at ERT start 50.7 years [range 23–69]; van der Ploeg et al. [18] mean age 45.3 years [range 15.9–70.0]; Ravaglia et al. [19] mean age 54.2 years; and Wokke et al. [7] median age 42.6 years [range 24.3–68.5]). When evaluating the effect of a treatment in a chronic disease such as late-onset Pompe disease it is important to take into account the natural decline in function that occurs with age, as well as the deleterious impact of the disease. In this context, stabilization of muscle function may reflect a positive impact of treatment on disease progression in late-onset Pompe disease. In older late-onset Pompe patients, however, stabilization may not occur during ERT because of the natural age-related decline in walking ability, although the rate of decline may be attenuated; long-term studies are required to evaluate this.

Although it is a valuable and widely used functional measure, the 6MWT is associated with considerable inter- and intra-investigator variability [17], and does not elucidate the mechanisms behind the compromised physical function that it measures [20]. In addition, the distance walked can be affected by factors such as patient motivation, age, sex, height, and weight as well as skeletal problems [16], which can affect gait and thereby influence the distance walked. Moreover, a high proportion of late-onset Pompe patients become wheelchair-dependent over time and so the 6MWT is no longer relevant. This is an important consideration for long-term clinical studies.

FVC provides a simple measure of pulmonary function and is a widely used outcome measure in studies of patients with NMDs. However, the presence of factors such as severe scoliosis or other lung disease can impact on the reliability of results [21].

### Clinical relevance of the 6MWT and FVC

#### 6MWT: MCID

Nine studies [22-30] were identified in chronic diseases other than late-onset Pompe disease that had aimed to relate changes in the 6MWT to changes in patient perception (Table 1) [12-14,18,19,22-35]. Redelmeier and colleagues found that in a cohort of 112 patients with COPD, the 6MWD would need to differ by 54 m (95% CI 37–71 m), a relative change of 15%, for patients to stop perceiving their walking ability as “about the same” and to begin to rate themselves as “a little bit better” [22]. Studies in diseases such as IPF [23], coronary artery disease following acute coronary syndrome [24], and pulmonary arterial hypertension [25] identified a 5–11% relative change from baseline as the minimal clinically important change or MCID for the 6MWT (Table 1).

**Table 1:** Clinical relevance of 6MWD changes in patients with chronic respiratory disease and late-onset Pompe disease

**Source**	**Disease**	**n**	**6MWT**				**Relevance**			**Authors’ conclusions**
			**Mean ± SD baseline (m)**	**Timepoint (m)**	**Absolute diff. (m)**	**Relative diff. (%)**	Absolute MCID* (m)	**Relative MCID**^**† **^**(%)**	**Patient notice change?**^**‡**^	
[22]	COPD	112	371 ± 129				54	15		“Awareness of the smallest difference in walking distance that is noticeable to patients may help clinicians interpret effectiveness of treatments”
[23]	IPF	826	392 ± 109				24–45	6–11		“6MWT is a reliable, valid and responsive measure of exercise tolerance in IPF”
[24]	CAD after ACS	81	~480	Week 8: 553	73 ± 57	15	25	5	“same” to “a little bit better”	“A MCID of 25 m will help practitioners interpret changes in 6MWD in patients with CAD after ACS”
[25]	PAH	405	343 ± 77				~33	10		“The MID of 33 m helps assess treatment responses during trials of specific PAH therapies and sample size calculations”
[26]	COPD	460	361 ± 112				35	10		“The low correlations between 6MWT and patient-reported anchors questions whether a minimal important difference exists”
[27]	DMD	18	357	1 year: 300	−57	16	Above	Above	No	“The 6MWD changes at 1 year confirm the validity of this endpoint and emphasize that preserving ambulation must remain a major goal of DMD therapy”
[28]	DMD	21	366 ± 83	1 week: 364 ± 87	−2	<1	Below	Below	No	“Modified 6MWT is feasible and safe, documents disease-related limitations on ambulation, is reproducible, and offers a new outcome measure for DMD natural history and therapeutic trials”
[29]	PAH	213	330 ± 74	Week 16: 366	36	11	Within	Above	No	“Treatment increased the time to clinical worsening”
[30]	MPS	22	319 ± 131	Week 26: 339 ± 127	20	6	Below	Within	No	“Treatment translated into clinically important improvements in physical capacity (6MWT)”
[18]	Pompe	60	332 ± 127	Week 78: 358 ± 141	Week 78: 25	8	Within	Within	No	
[14]	Pompe	5	64	3 years: 184	120	188	Above	Above	“same” to “somewhat better”	
[12]	Pompe	58	320 ± 161	1–3 years: 383 ± 178	63	20	Above	Above	“same” to “a little bit better”	
[31]	Pompe	22	341 ± 150	1 year: 393 ± 157	52	15	Within	Above	“same” to “a little bit better”	
[13]	Pompe	21	312 ± 166	3 years: 326 ± 175	14	5	Below	Within	No	
[32]	Pompe	17	117 (median)	3 years: 265	148	126	Above	Above	“same” to “somewhat better”	
[19]	Pompe	11	246 ± 185	1.5–2 years: 295 ± 195	49	20	Within	Above	“same” to “a little bit better”	
[33]	Pompe	1	320	4 months: 500	180	56	Above	Above	“same” to “much better”	
[34]	Pompe	1	~375	32 months: 353	−44	11	Within	Above	No	
[35]	Pompe	2	1 pt: 660	6 months: 700	40	6	Within	Within	Maybe “same” to “a little bit better”	

*Absolute MCID defined as 24–54 m based on the range described by Redelmeier et al. [22] and du Bois et al. [23].

^†^Relative MCID defined as 5–10% based on range described by du Bois et al. [23], Gremeaux et al. [24], Mathai et al. [25], and Puhan et al. [26].

^**‡**^According to the publication by Redelmeier et al. [22] in patients with COPD, patients change their rating from “same” to “a little bit better” with an increase of 40 m in the 6MWT; from “same” to “somewhat better” with an increase of 100 m; and from “same” to “much better” with an increase of approximately 180 m. For a patient to rate the change from “same” to “a little bit worse”, the decrease in the 6MWT needs to be 70 m.

*ACS* acute coronary syndrome, *CAD* coronary artery disease, *COPD* chronic obstructive pulmonary disease, *diff.* difference, *DMD* Duchenne muscular dystrophy, *IPF* idiopathic pulmonary fibrosis, *MCID* minimal clinically important difference, *MPS* mucopolysaccharidosis I, *6MWD* 6MWT distance, *6MWT* 6-minute walking test, *PAH* pulmonary arterial hypertension, *pt* patient, *SD* standard deviation.

**Figure 1:**
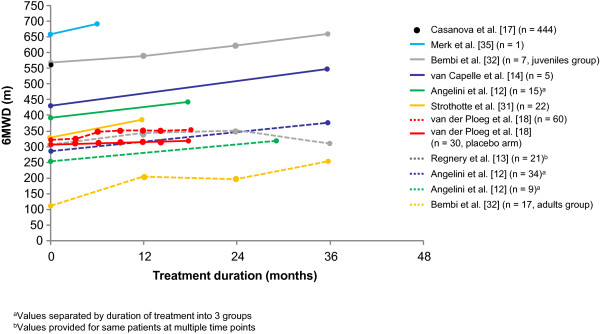
**6MWD in healthy adults and late-onset Pompe patients (untreated and those receiving treatment) [**[37]**].** 6MWD in healthy adults (black dot) [17], untreated late-onset Pompe patients (red line, data extracted from van der Ploeg et al. [18]), and across several studies of Pompe patients receiving alglucosidase alfa treatment [12-14,18,31,32,35]. Adapted with permission from [37] © 2013, Springer Science + Business Media. 6MWD, 6-minute walking test distance.

Studies of the natural history of untreated late-onset Pompe disease [5,7,36] have not reported changes in walking distance as observational data. However, a randomized study of ERT in late-onset Pompe disease included data for a placebo group (30 patients) followed up for 18 months [18]. Here the mean decrease in walking distance was 3 m in untreated patients over 18 months (mean baseline 6MWD 317.9 m) (Figure 1) [12-14,17,18,31,32,35,37]. This is equivalent to a relative change of 0.6% annually in walking ability. Applying the 6MWD MCID to this untreated population, and assuming that the MCID for the 6MWD deterioration is the same as for improvement, it would take ≥9 years for patients to experience a clinically significant decrease in walking distance.

Ten clinical studies of late-onset Pompe patients treated with alglucosidase alfa (Myozyme^®^; Genzyme, Cambridge, MA, USA) reported the 6MWT as a functional outcome measure (Table 1) [12-14,18,19,31-35]. When the absolute and relative MCID for the 6MWT identified in other chronic diseases was applied to the findings of clinical trials in late-onset Pompe disease, the majority of studies (9 of 10) reported absolute changes from baseline in 6MWT that lay within [18,19,31,34,35] or above [12,14,32,33] the absolute MCID levels reported for other diseases (24–54 m). All 10 clinical studies reported a relative change from baseline that was above or within the MCID range (5–11%) established for other diseases [12-14,18,19,31-35]. These findings indicate that in late-onset Pompe disease ERT is associated with an improved functional capacity which, if observed in patients with respiratory disease, would be expected to manifest as either a disease stabilization or noticeable physical improvement for patients.

#### FVC: MCID

**Figure 2:**
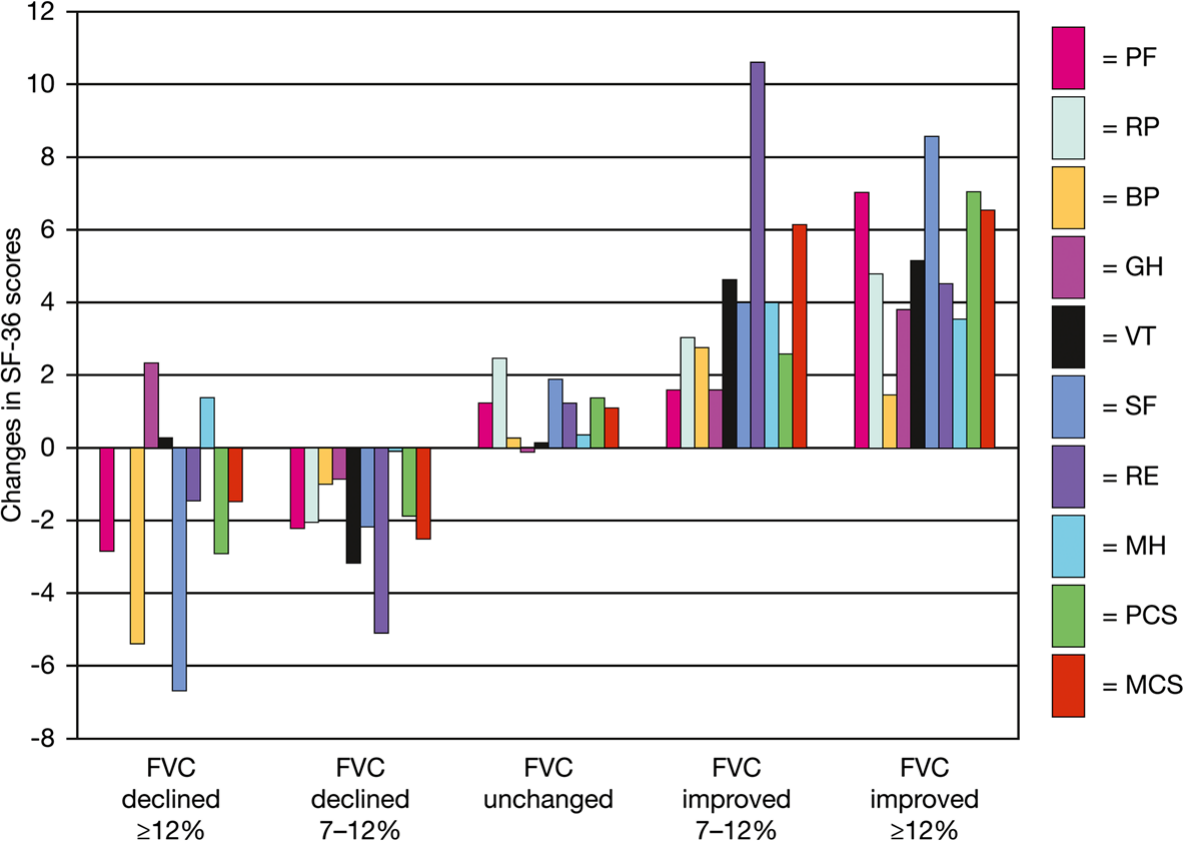
**Changes in SF-36 scores stratified by changes in FVC percentage in patients with IPF **[41]**.** Reproduced from [41] © 2010, with permission from Elsevier. BP, bodily pain; FVC, forced vital capacity; GH, general health; MCS, mental component summary; MH, mental health; PCS, physical component summary score; PF, physical functioning; RE, role emotional domain; RP, role physical; SF, social functioning; VT, vitality.

**Figure 3:**
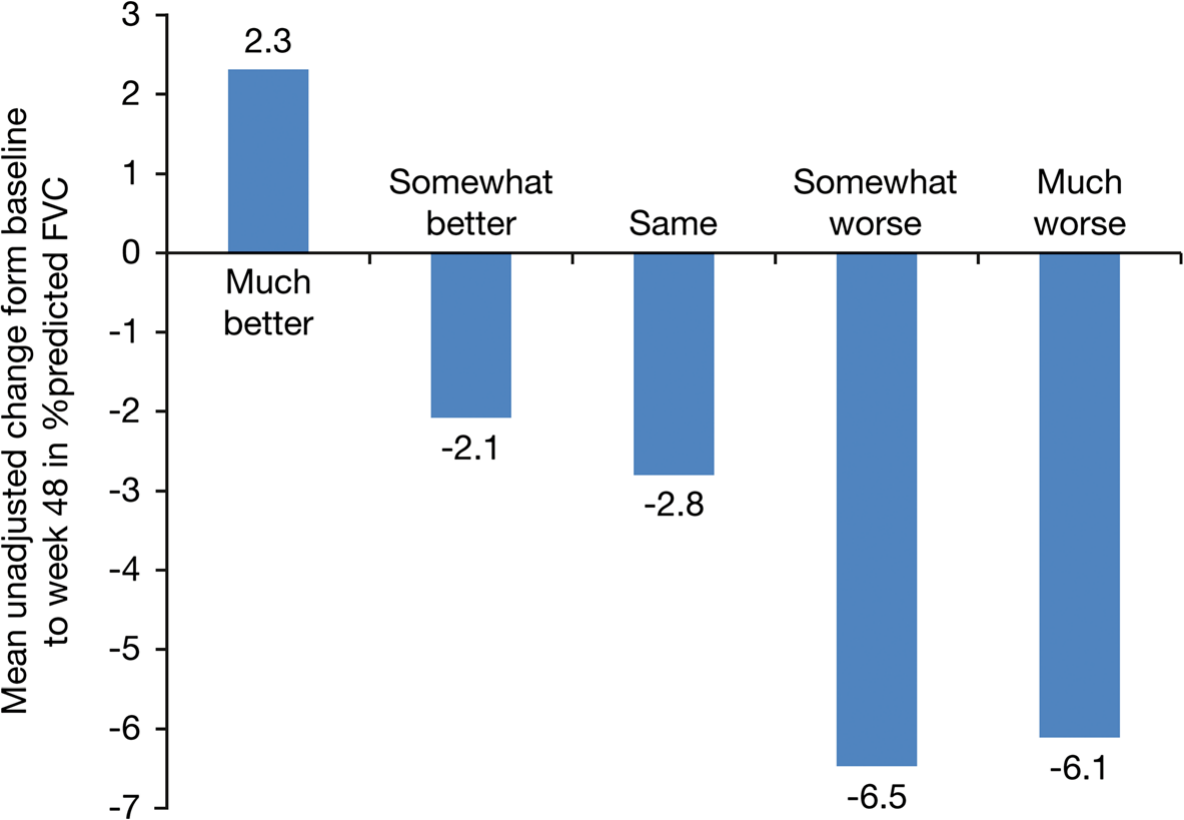
**Changes in global health status according to changes in % predicted FVC in patients with IPF [**[39]**].** FVC, forced vital capacity. IPF, idiopathic pulmonary fibrosis.

When FVC is used as a measure of respiratory function, predicted FVC values >80% are considered to be within normal range. In patients with chronic lung diseases, change in FVC over time is a valid outcome measure. Guidelines for the assessment of patients with systemic scleroderma cite that an improvement or reduction of 10% from baseline values is required to ensure that the variation in lung capacity can be ascribed to a change in disease severity rather than measurement error [38]. However, review of the studies identified here which used FVC as an outcome measure revealed that the definition of a relevant change from baseline in FVC is variable (Table 2) [12,13,18,30,31,34,39-45]. In 2 studies in patients with IPF, absolute changes of between 5 and 10% (equivalent to relative changes of 7–14%) at 6 months were considered “unchanged” or “marginal”; and “more than minimal” or “significant” changes in FVC were associated with absolute changes of >10–12% (equivalent to relative changes of >14–18%) [40,41]. Such changes were also found to impact on quality of life (QoL; Figure 2) [41]. In a larger study of 1156 patients with IPF [39], the MCID in FVC was defined as 2–6% (equivalent to a 3–9% relative change) and changes from baseline in % predicted FVC reflected changes in global health status (Figure 3) [39].

**Table 2:** Clinical relevance of FVC changes in patients with chronic respiratory disease and late-onset Pompe disease

**Citation**	**Disease**	**n**	**% predicted FVC**				Relevance*		
			**Mean ± SD baseline (m)**	**Timepoint (m)**	**Absolute diff. (%)**	**Relative diff. (%)**	**Absolute MCID (%)**	**Relative MCID (%)**	**Patient notice difference?**
[39]	IPF	1156	70 ± 13				2–6	3–9	
[40]	IPF	84	73 ± 19		5–10	7–14			
					>10	>14			
[41]	IPF		67 ± 12		7–12	10–18			
					≥12	≥18			
[30]	MPS	22	48 ± 15	Week 26: 53 ± 19	5	10	Within	Above	“much better”
[18]	Pompe	60	55 ± 14	Week 78: 57 ± 16	Week 78: 1	2	Below	Below	“much better” to “somewhat better”
[42]	Pompe	2	Pt 1: 81	2 years:	2 years:	2 years:	Pt 1: Below	Pt 1: Below	Pt 1: “much better” to “somewhat better”
			Pt 2: 94	Pt 1: 80	Pt 1: −1	Pt 1: −1	Pt 2: Within	Pt 2: Below	Pt 2: “much better” to “somewhat better”
				Pt 2: 92	Pt 2: −2	Pt 2: −2			
[12]	Pompe	74	65 ± 27	67 ± 27	2	3	Within	Within	“much better” to “somewhat better”
[31]	Pompe	44	70	12 months: 70	12 months: 0.5	1	Below	Below	“much better” to “somewhat better”
[13]	Pompe	28	80 ± 14	At 3 years: 77 ± 18	3 years: −4	5	Within	Within	“same” to “much worse”
[43]	Pompe	1	44	42	−2	−5	Within	Within	“somewhat better”
			----------	----------	----------	----------	----------	----------	----------
			31	25	−6	−19	Above	Above	“same” to “much worse”
[44]	Pompe	5		2 years:	2 years:				
			Pt 1: 0.0	Pt 1: 8	Pt 1: 8	Pt 1: -	Pt 1: Above	Pt 1: -	Pt 1: “much better”
			Pt 2: 46	Pt 2: 66	Pt 2: 26	Pt 2: 57	Pt 2: Above	Pt 2: Above	Pt 2: “much better”
			Pt 3: 9	Pt 3: 16	Pt 3: 7	Pt 3: 78	Pt 3: Above	Pt 3: Above	Pt 3: “much better”
			Pt 4: 14	Pt 4: 7	Pt 4: −7	Pt 4: 50	Pt 4: Above	Pt 4: Above	Pt 4: “somewhat worse”
			Pt 5: 10	Pt 5: 20	Pt 5: 10	Pt 5: 100	Pt 5: Above	Pt 5: Above	Pt 5: “much better”
[34]	Pompe	1			1.9/year		Below		“much better” to “somewhat better”
[45]	Pompe	1			6 months: 16		Above		“much better”

*MCID of 2–6% and patient classifications based on a study in patients with idiopathic pulmonary fibrosis [39].

*FVC* forced vital capacity, *IPF* idiopathic pulmonary fibrosis, *MCID* minimal clinically important difference, *pt* patient, *diff.* difference, *% predicted FVC* Percentage change in predicted FVC, *MPS* mucopolysaccharidosis, *SD* standard deviation.

Evidence from studies in patients with untreated late-onset Pompe disease indicate that the expected change in % predicted FVC in the upright position and in the supine position range from −1.0% to −4.6% [5,7,36,46] and 1.3% to −5.5% [5,7,36], respectively, annually. At the rate of disease progression observed in untreated patients with late-onset Pompe disease, and assuming that the MCID for deterioration of the FVC is the same as for improvement in FVC reported in IPF patients, untreated late-onset Pompe patients would have clinically significant worsening of lung function within 2 years.

Nine clinical studies of late-onset Pompe patients were identified that used % predicted FVC as an outcome measure [12,13,18,31,34,42-45]. When the MCID ranges established for FVC in IPF were applied to these studies, both the absolute and relative change in FVC were above the MCID range in 2 studies [44,45], within the MCID in 3 studies [12,13,43], and below the MCID in 4 studies (Table 2) [18,31,34,42]. In the majority of studies of alglucosidase alfa treatment, patients who had a change in FVC within or above the MCID range established for IPF also reported a noticeable improvement. However, there were some studies in which the change observed was below the MCID, but the patients still reported feeling either “somewhat better” or “much better” in their overall health [18,31,34]. This correlates with findings reported in a study in IPF in which patients whose mean % predicted FVC declined by 2.1% nevertheless reported that their global health status was “somewhat better” (Figure 3) [39].

#### Limitations of applying MCID from other chronic diseases to late-onset Pompe disease

It is important to note that applying the MCID for the 6MWT and FVC established in studies of chronic respiratory diseases to late-onset Pompe studies has several important limitations. The IPF and late-onset Pompe studies with measure disease course in patients with a different set of pathologies and symptoms. For example, in pulmonary fibrosis, FVC reflects lung volumes and airway obstruction, not respiratory effort. Also, in respiratory diseases, QoL relates to breathlessness more than to muscle function. In this analysis of the clinical relevance of 6MWD in late-onset Pompe disease, patients perceived an improvement in their walking ability in 7 of the 10 studies identified [12,14,19,31-33,35], despite the fact that in the 3 remaining studies [13,18,34], the absolute changes in 6MWD were below/within, and the relative changes in 6MWD were within/above, the MCID range extrapolated from studies of other diseases. This may exemplify the problems of applying data from different diseases (e.g. COPD, IPF) to late-onset Pompe patients and highlights the need to explore alternative clinical endpoints for long-term clinical studies in these patients.

### Outcome measures currently used in patients with DMD/BMD

The clinical presentation and symptoms of late-onset Pompe disease are similar to many of the NMDs, particularly DMD/BMD. Therefore, it is instructive to look at the measures of strength and function that have been used to assess progressive muscle weakness in studies of patients with other NMDs. A wide range of outcome measures have been studied in DMD, including the 6MWT and other timed functional tests (e.g. timed stair climb, time to get up from the floor) [27,47-51]. Although timed functional activities are reliable outcome measures in DMD [49], the clinical relevance of changes measured using these methods and the correlation with day-to-day activities and QoL in DMD remains to be established. In many cases the standard tests may benefit from modification to reflect disease-specific factors; for example the 6MWT has been modified to suit the needs of patients with DMD [28].

Validated disease-specific outcome measures have been developed to assess the clinical relevance of changes in physical function during treatment of DMD. The multicomponent North Star Ambulatory Assessment (NSAA) assesses functional ability in boys with DMD [52-54], and includes the 6MWT and several other timed muscle function tests [54]. NSAA has proved to be a reliable and valid measure of walking ability in boys with DMD, as assessed by Rasch analysis [53], and it has been shown to be responsive to treatment-related change over time, supporting its use as an endpoint in clinical trials [55]. Another composite assessment recently developed for use in the study of DMD and other NMDs is the Motor Function Measure (MFM), which evaluates 3 different dimensions of motor performance. The MFM has good responsiveness in patients with DMD and good correlation with patient and physician perceptions of clinical changes [56].

Recent studies in DMD patients have shown that the use of a combination of outcome measures (e.g. NSAA, 6MWT, timed muscle function tests) are an effective approach that could provide information on different aspects of motor function which may not be detected by a single measure [54]. Using a similar approach, the Cooperative International Neuromuscular Research Group (CINRG) has developed a standardized composite muscle strength testing system — the CINRG Quantitative Measurement System (CQMS) — which combines anthropometrics and muscle, pulmonary, functional, and timed tests [57]. The CQMS is currently being evaluated in patients with DMD (ClinicalTrials.gov identifier, NCT01125709).

Progressive neuromuscular impairment has a significant impact on health-related QoL and limits ADL in patients with muscular dystrophy. This can be assessed using questionnaires such as the generic Dutch instruments TACQOL (for children aged 6–15 years) and TAAQOL (for adults) [58], and the ACTIVLIM questionnaire for ADL [59]. Interestingly, motor impairments in patients with NMDs do not correlate well with measures of activity limitation [59].

### Outcome measures used in neuromuscular and other chronic diseases

Measurement of walking speed is a quick and inexpensive test, which has been shown to be predictive of survival in older adults [60,61]. Walking speed reflects a range of factors such as energy, movement control and support, as well as the strength of cardiovascular, pulmonary, and musculoskeletal systems [60]; it may also be of value in assessing disease progression or treatment effect in NMDs. Several performance tests are used to assess walking speed in the clinical environment. In patients with amyotrophic lateral sclerosis (ALS) the 10 m walking (gait) speed is a validated and reliable measure of disease progression [62], and the timed ‘get up and go’ test has been validated in patients with multiple sclerosis (MS) and used to measure functional mobility [63].

Ideally, walking speed should be measured in an uncontrolled setting that more closely reflects real-life activity. This is now being made possible by advances in mobile accelerometry. For example, actibelt^®^, a 3D accelerometer which can be placed inside a belt buckle, has been developed to calculate walking speed [64]. Recent data from patients with MS suggest that although the actibelt^®^ may be useful for measuring walking speed in patients with mild functional disabilities it was less precise than the 6MWT, and accuracy worsened in patients with more severe walking impairments [65]. Temporal and spatial parameters of gait can be measured using the GAITRite^®^ Walkway System, an electronic pressure-sensitive walkway. GAITRite^®^ scores have been shown to be sensitive to disease-related gait changes in patients with mucopolysaccharidosis [66] and Wolfram Syndrome [67]. In patients with MS, changes in temporo-spatial gait parameters measured using GAITRite^®^ technology correlate with patient-reported changes in walking impairment as measured using the MS walking scale-12 [68]. However, a possible shortcoming is that the use of measures of walking speed and gait parameters is limited to patients with mild and moderate disability.

Disease-specific tools developed for measuring QoL/ADL in MS and ALS patients [69,70] might also be applicable to late-onset Pompe patients.

### Future directions: improving clinical endpoints for clinical trials in late-onset Pompe disease

Although not yet evaluated in late-onset Pompe disease, the sniff nasal inspiratory pressure (SNIP) test allows earlier detection of changes in respiratory muscle strength than FVC, and has been shown to be reliable and easy for young DMD patients to perform [71]. SNIP might also be a useful measure to assess late-onset Pompe patients who have bulbar muscle involvement [72]. MIP and MEP are direct measures of respiratory function that have also been used as outcome measures in clinical studies of late-onset Pompe disease treatment [18,72]. MIP and MEP reflect neuromuscular (diaphragmatic) ventilation dysfunction and may, therefore, better discriminate progressive muscular weakness from interstitial lung function alterations such as those seen in fibrotic lung disease. A recently published retrospective analysis of data from adults with late-onset Pompe disease found that although upright vital capacity measurement provides a strong measure that can be used for long-term follow-up of respiratory function, MEP and maximal SNIP also correlated with lung and respiratory function as measured using non-invasive transdiaphragmatic methods [73]. However, the clinical relevance of MIP and MEP as endpoints in clinical studies still requires validation. The hours of use of home mechanical ventilation (HMV) per day and the number of hospitalizations necessary due to pulmonary exacerbations were more sensitive indicators than FVC of variations in breathing autonomy in response to long-term ERT, according to a study in late-onset Pompe patients with severe pulmonary impairment [74]. Therefore, these indicators might be considered as alternative respiratory outcomes in late-onset Pompe patients with high ventilator dependency [74].

The Gait, Stairs, Chair, Gower (GSCG) score has recently been validated as an alternative outcome measure for motor function in late-onset Pompe patients receiving ERT [75]. The GSCG score is a composite test that evaluates the four main motor performances quantitatively and qualitatively and should be used in combination with both the WGM and the 6MWT to identify individual response to ERT in late-onset Pompe patients [75].

Inter- and intra-investigator variances of measurements in non-technically guided investigations should also be considered when designing new clinical trials. In this context, technically-assisted measurements may overcome the intra- and inter-investigator differences in outcome measures associated with endpoints such the 6MWT, and improve the reliability and validity of functional measurements.

Regulatory authorities are increasingly calling for outcome measures to be validated, reliable, and time-responsive. Assessments that are designed and tested using modern psychometric methods, such as Rasch analysis, meet these criteria. Recently a patient-based interval scale, the Rasch-designed Pompe-specific Activity (R-PAct) scale, has been developed and validated [76]. The R-PAct scale detects limitations in physical activities and social participation throughout the entire disease spectrum in late-onset Pompe patients aged >16 years. As a measure of disease-specific patient-relevant outcomes, the R-PAct scale may have considerable potential as an endpoint in future clinical trials (e.g. phase IV studies of ERT in late-onset Pompe disease).

## Conclusions

To date, clinical trials in late-onset Pompe disease have commonly used endpoints such as the 6MWT and FVC to assess muscular and respiratory function during disease progression or treatment. Although there is evidence of the clinical relevance of these endpoints in other chronic progressive illnesses, it is important to establish that these parameters are reliable, valid, responsive, and clinically relevant for late-onset Pompe patients. Our analysis identified the range of MCID (absolute and relative) for 6MWT and FVC in chronic respiratory disease, and extrapolated these findings to the natural history of late-onset Pompe disease and to the results of clinical studies of ERT in late-onset Pompe disease. Our data suggest that in untreated late-onset Pompe patients a MCID deterioration in FVC would occur in approximately 2 years and a deterioration in the 6MWD within 9 years. In a majority of studies in which late-onset Pompe patients were treated with alglucosidase alfa, the changes from baseline in the 6MWT were above or within the MCID established in respiratory diseases, and a clinical improvement was perceived by patients in 7 of the 10 studies. Additionally, in two-thirds of the studies in which late-onset Pompe patients were treated with alglucosidase alfa, the changes from baseline in % predicted FVC were above or within the MCID established in respiratory diseases, and the difference was perceived as either an improvement or stabilization by patients. These data suggest that the changes observed during alglucosidase alfa treatment could represent significant reversal of the disease, rather than just stabilization, particularly when we consider the relatively short duration (up to 3 years) of the late-onset Pompe studies included in our analysis. However, longer-term studies are required to confirm this hypothesis. Furthermore, as the relevance of 6MWT or FVC MCIDs to late-onset Pompe patients is unclear, future studies evaluating the MCID of these two measures in these patients are also required, as well as studies evaluating the clinical value of potential alternative outcome measures. Attention should be given to identifying and validating alternative endpoints (e.g. SNIP, MIP, MEP, R-PAct) for use in future clinical trials, as well as late-onset Pompe disease-specific questionnaires addressing QoL and ADL. Such measures may be more reflective of the challenges faced by late-onset Pompe patients in their day-to-day life and have greater long-term clinical relevance.

## Abbreviations

ADL: Activities of daily living; ALS: Amyotrophic lateral sclerosis; BMD: Becker muscular dystrophy; CINRG: Cooperative international neuromuscular research group; COPD: Chronic obstructive pulmonary disease; CQMS: CINRG quantitative measurement system; DMD: Duchenne muscular dystrophy; ERT: Enzyme replacement therapy; FVC: Forced vital capacity; HMV: Home mechanical ventilation; IPF: Idiopathic pulmonary fibrosis; MCID: Minimal clinically important difference; MEP: Maximum expiratory pressure; MFM: Motor function measure; MIP: Maximum inspiratory pressure; MS: Multiple sclerosis; NMD: Neuromuscular disorders; NSAA: North star ambulatory assessment; QoL: Quality of life; R-PAct: Rasch-designed Pompe-specific activity; SNIP: Sniff nasal inspiratory pressure; WGM: Walton Gardner Medwin; % predicted FVC: Percentage change in predicted FVC; 6MWD: 6MWT distance; 6MWT: 6-minute walking test.

## Competing interests

RL has received honoraria for invited lectures from Genzyme, a Sanofi Company. BS is member of the Pompe Global Advisory Board, sustained by Genzyme, a Sanofi Company, and has received honoraria for consultation and invited lectures.

## Authors’ contributions

RL and BS have contributed to conception and drafting of the manuscript and interpretation of data. All authors read and approved the final manuscript.

## References

[B1] Ausems MG Verbiest J Hermans MP Kroos MA Beemer FA Wokke JH Sandkuijl LA Reuser AJ van der Ploeg AT Frequency of glycogen storage disease type II in the Netherlands: implications for diagnosis and genetic counselling Eur J Hum Genet 1999 8 713 716 10.1038/sj.ejhg.5200367 10482961

[B2] Chien YH Chiang SC Zhang XK Keutzer J Lee NC Huang AC Chen CA Wu MH Huang PH Tsai FJ Chen YT Hwu WL Early detection of Pompe disease by newborn screening is feasible: results from the Taiwan screening program Pediatrics 2008 8 e39 e45 10.1542/peds.2007-2222 18519449

[B3] Martiniuk F Chen A Mack A Arvanitopoulos E Chen Y Rom WN Codd WJ Hanna B Alcabes P Raben N Plotz P Carrier frequency for glycogen storage disease type II in New York and estimates of affected individuals born with the disease Am J Med Genet 1998 8 69 72 10.1002/(SICI)1096-8628(19980827)79:1<69::AID-AJMG16>3.0.CO;2-K 9738873

[B4] Schüller A Wenninger S Strigl-Pill N Schoser B Toward deconstructing the phenotype of late-onset Pompe disease Am J Med Genet C Semin Med Genet 2012 8 80 88 22253010 10.1002/ajmg.c.31322

[B5] van der Beek NA de Vries JM Hagemans ML Hop WC Kroos MA Wokke JH de Visser M van Engelen BG Kuks JB van der Kooi AJ Notermans NC Faber KG Verschuuren JJ Reuser AJ van der Ploeg AT van Doorn PA Clinical features and predictors for disease natural progression in adults with Pompe disease: a nationwide prospective observational study Orphanet J Rare Dis 2012 8 88 10.1186/1750-1172-7-88 23147228 PMC3551719

[B6] Müller-Felber W Horvath R Gempel K Podskarbi T Shin Y Pongratz D Walter MC Baethmann M Schlotter-Weigel B Lochmüller H Schoser B Late onset Pompe disease: clinical and neurophysiological spectrum of 38 patients including long-term follow-up in 18 patients Neuromuscul Disord 2007 8 698 706 10.1016/j.nmd.2007.06.002 17643989

[B7] Wokke JH Escolar DM Pestronk A Jaffe KM Carter GT van den Berg LH Florence JM Mayhew J Skrinar A Corzo D Laforet P Clinical features of late-onset Pompe disease: a prospective cohort study Muscle Nerve 2008 8 1236 1245 10.1002/mus.21025 18816591

[B8] Güngör D de Vries JM Hop WC Reuser AJ van Doorn PA van der Ploeg AT Hagemans ML Survival and associated factors in 268 adults with Pompe disease prior to treatment with enzyme replacement therapy Orphanet J Rare Dis 2011 8 34 10.1186/1750-1172-6-34 21631931 PMC3135500

[B9] Winkel LP Hagemans ML van Doorn PA Loonen MC Hop WJ Reuser AJ van der Ploeg AT The natural course of non-classic Pompe’s disease; a review of 225 published cases J Neurol 2005 8 875 884 10.1007/s00415-005-0922-9 16133732

[B10] Güngör D Kruijshaar ME Plug I D’Agostino RB Sr Hagemans ML van Doorn PA Reuser AJ van der Ploeg AT Impact of enzyme replacement therapy on survival in adults with Pompe disease: results from a prospective international observational study Orphanet J Rare Dis 2013 8 49 10.1186/1750-1172-8-49 23531252 PMC3623847

[B11] Schneider I Hanisch F Müller T Schmidt B Zierz S Respiratory function in late-onset Pompe disease patients receiving long-term enzyme replacement therapy for more than 48 months Wien Med Wochenschr 2013 8 40 44 10.1007/s10354-012-0153-5 23160972

[B12] Angelini C Semplicini C Ravaglia S Bembi B Servidei S Pegoraro E Moggio M Filosto M Sette E Crescimanno G Tonin P Parini R Morandi L Marrosu G Greco G Musumeci O Di Iorio G Siciliano G Donati MA Carubbi F Ermani M Mongini T Toscano A Italian GSDII Group Observational clinical study in juvenile-adult glycogenosis type 2 patients undergoing enzyme replacement therapy for up to 4 years J Neurol 2012 8 952 958 10.1007/s00415-011-6293-5 22081099

[B13] Regnery C Kornblum C Hanisch F Vielhaber S Strigl-Pill N Grunert B Müller-Felber W Glocker FX Spranger M Deschauer M Mengel E Schoser B 36 Months observational clinical study of 38 adult Pompe disease patients under alglucosidase alfa enzyme replacement therapy J Inherit Metab Dis 2012 8 837 845 10.1007/s10545-012-9451-8 22290025

[B14] van Capelle CI van der Beek NA Hagemans ML Arts WF Hop WC Lee P Jaeken J Frohn-Mulder IM Merkus PJ Corzo D Puga AC Reuser AJ van der Ploeg AT Effect of enzyme therapy in juvenile patients with Pompe disease: a three-year open-label study Neuromuscul Disord 2010 8 775 782 10.1016/j.nmd.2010.07.277 20817528

[B15] van der Ploeg AT Barohn R Carlson L Charrow J Clemens PR Hopkin RJ Kishnani PS Laforêt P Morgan C Nations S Pestronk A Plotkin H Rosenbloom BE Sims KB Tsao E Open-label extension study following the Late-Onset Treatment Study (LOTS) of alglucosidase Alfa Mol Genet Metab 2012 8 456 461 10.1016/j.ymgme.2012.09.015 23031366

[B16] ATS Committee on Proficiency Standards for Clinical Pulmonary Function Laboratories ATS statement: guidelines for the six-minute walk test Am J Respir Crit Care Med 2002 8 111 117 12091180 10.1164/ajrccm.166.1.at1102

[B17] Casanova C Celli BR Barria P Casas A Cote C de Torres JP Jardim J Lopez MV Marin JM Montes de Oca M Pinto-Plata V Aguirre-Jaime A Six Minute Walk Distance Project (ALAT) The 6-min walk distance in healthy subjects: reference standards from seven countries Eur Respir J 2011 8 150 156 10.1183/09031936.00194909 20525717

[B18] van der Ploeg AT Clemens PR Corzo D Escolar DM Florence J Groeneveld GJ Herson S Kishnani PS Laforet P Lake SL Lange DJ Leshner RT Mayhew JE Morgan C Nozaki K Park DJ Pestronk A Rosenbloom B Skrinar A van Capelle CI van der Beek NA Wasserstein M Zivkovic SA A randomized study of alglucosidase alfa in late-onset Pompe’s disease N Engl J Med 2010 8 1396 1406 10.1056/NEJMoa0909859 20393176

[B19] Ravaglia S Pichiecchio A Ponzio M Danesino C Saeidi Garaghani K Poloni GU Toscano A Moglia A Carlucci A Bini P Ceroni M Bastianello S Changes in skeletal muscle qualities during enzyme replacement therapy in late-onset type II glycogenosis: temporal and spatial pattern of mass vs. strength response J Inherit Metab Dis 2010 8 737 745 10.1007/s10545-010-9204-5 20844963

[B20] Heresi GA Dweik RA Strengths and limitations of the six-minute-walk test: a model biomarker study in idiopathic pulmonary fibrosis Am J Respir Crit Care Med 2011 8 1122 1124 10.1164/rccm.201012-2079ED 21531951

[B21] Inal-Ince D Savci S Arikan H Saglam M Vardar-Yagli N Bosnak-Guclu M Dogru D Effects of scoliosis on respiratory muscle strength in patients with neuromuscular disorders Spine J 2009 8 981 986 10.1016/j.spinee.2009.08.451 19819188

[B22] Redelmeier DA Bayoumi AM Goldstein RS Guyatt GH Interpreting small differences in functional status: the Six Minute Walk Test in chronic lung disease patients Am J Respir Crit Care Med 1997 8 1278 1282 10.1164/ajrccm.155.4.9105067 9105067

[B23] du Bois RM Weycker D Albera C Bradford WZ Costabel U Kartashov A Lancaster L Noble PW Sahn SA Szwarcberg J Thomeer M Valeyre D King TE Jr Six-minute-walk test in idiopathic pulmonary fibrosis: test validation and minimal clinically important difference Am J Respir Crit Care Med 2011 8 1231 1237 10.1164/rccm.201007-1179OC 21131468

[B24] Gremeaux V Troisgros O Benaïm S Hannequin A Laurent Y Casillas JM Benaïm C Determining the minimal clinically important difference for the six-minute walk test and the 200-meter fast-walk test during cardiac rehabilitation program in coronary artery disease patients after acute coronary syndrome Arch Phys Med Rehabil 2011 8 611 619 10.1016/j.apmr.2010.11.023 21440707

[B25] Mathai SC Puhan MA Lam D Wise RA The minimal important difference in the 6-minute walk test for patients with pulmonary arterial hypertension Am J Respir Crit Care Med 2012 8 428 433 10.1164/rccm.201203-0480OC 22723290 PMC3443803

[B26] Puhan MA Mador MJ Held U Goldstein R Guyatt GH Schünemann HJ Interpretation of treatment changes in 6-minute walk distance in patients with COPD Eur Respir J 2008 8 637 643 10.1183/09031936.00140507 18550610

[B27] McDonald CM Henricson EK Han JJ Abresch RT Nicorici A Atkinson L Elfring GL Reha A Miller LL The 6-minute walk test in Duchenne/Becker muscular dystrophy: longitudinal observations Muscle Nerve 2010 8 966 974 10.1002/mus.21808 21038378

[B28] McDonald CM Henricson EK Han JJ Abresch RT Nicorici A Elfring GL Atkinson L Reha A Hirawat S Miller LL The 6-minute walk test as a new outcome measure in Duchenne muscular dystrophy Muscle Nerve 2010 8 500 510 10.1002/mus.21544 19941337

[B29] Rubin LJ Badesch DB Barst RJ Galie N Black CM Keogh A Pulido T Frost A Roux S Leconte I Landzberg M Simonneau G Bosentan therapy for pulmonary arterial hypertension N Engl J Med 2002 8 896 903 10.1056/NEJMoa012212 11907289

[B30] Wraith JE Clarke LA Beck M Kolodny EH Pastores GM Muenzer J Rapoport DM Berger KI Swiedler SJ Kakkis ED Braakman T Chadbourne E Walton-Bowen K Cox GF Enzyme replacement therapy for mucopolysaccharidosis I: a randomized, double-blinded, placebo-controlled, multinational study of recombinant human alpha-L-iduronidase (laronidase) J Pediatr 2004 8 581 588 10.1016/j.jpeds.2004.01.046 15126990

[B31] Strothotte S Strigl-Pill N Grunert B Kornblum C Eger K Wessig C Deschauer M Breunig F Glocker FX Vielhaber S Brejova A Hilz M Reiners K Müller-Felber W Mengel E Spranger M Schoser B Enzyme replacement therapy with alglucosidase alfa in 44 patients with late-onset glycogen storage disease type 2: 12-month results of an observational clinical trial J Neurol 2010 8 91 97 10.1007/s00415-009-5275-3 19649685

[B32] Bembi B Pisa FE Confalonieri M Ciana G Fiumara A Parini R Rigoldi M Moglia A Costa A Carlucci A Danesino C Pittis MG Dardis A Ravaglia S Long-term observational, non-randomized study of enzyme replacement therapy in late-onset glycogenosis type II J Inherit Metab Dis 2010 8 727 735 10.1007/s10545-010-9201-8 20838899

[B33] Ishigaki K Murakami T Nakanishi T Oda E Sato T Osawa M Close monitoring of initial enzyme replacement therapy in a patient with childhood-onset Pompe disease Brain Dev 2012 8 98 102 10.1016/j.braindev.2011.05.004 21676566

[B34] de Vries JM van der Beek NA Kroos MA Ozkan L van Doorn PA Richards SM Sung CC Brugma JD Zandbergen AA van der Ploeg AT Reuser AJ High antibody titer in an adult with Pompe disease affects treatment with alglucosidase alfa Mol Genet Metab 2010 8 338 345 10.1016/j.ymgme.2010.08.009 20826098

[B35] Merk T Wibmer T Schumann C Krüger S Glycogen storage disease type II (Pompe disease)-influence of enzyme replacement therapy in adults Eur J Neurol 2009 8 274 277 10.1111/j.1468-1331.2008.02377.x 19138339

[B36] de Vries JM van der Beek NA Hop WC Karstens FP Wokke JH de Visser M van Engelen BG Kuks JB van der Kooi AJ Notermans NC Faber CG Verschuuren JJ Kruijshaar ME Reuser AJ van Doorn PA van der Ploeg AT Effect of enzyme therapy and prognostic factors in 69 adults with Pompe disease: an open-label single-center study Orphanet J Rare Dis 2012 8 73 10.1186/1750-1172-7-73 23013746 PMC3519647

[B37] Toscano A Schoser B Enzyme replacement therapy in late-onset Pompe disease: a systematic literature review J Neurol 2013 8 951 959 10.1007/s00415-012-6636-x 22926164

[B38] Wells AU Behr J Silver R Outcome measures in the lung Rheumatology (Oxford) 2008 8 Suppl 5 v48 v50 18784144 10.1093/rheumatology/ken311

[B39] du Bois RM Weycker D Albera C Bradford WZ Costabel U Kartashov A King TE Jr Lancaster L Noble PW Sahn SA Thomeer M Valeyre D Wells AU Forced vital capacity in patients with idiopathic pulmonary fibrosis: test properties and minimal clinically important difference Am J Respir Crit Care Med 2011 8 1382 1389 10.1164/rccm.201105-0840OC 21940789

[B40] Zappala CJ Latsi PI Nicholson AG Colby TV Cramer D Renzoni EA Hansell DM du Bois RM Wells AU Marginal decline in forced vital capacity is associated with a poor outcome in idiopathic pulmonary fibrosis Eur Respir J 2010 8 830 836 10.1183/09031936.00155108 19840957

[B41] Swigris JJ Brown KK Behr J du Bois RM King TE Raghu G Wamboldt FS The SF-36 and SGRQ: validity and first look at minimum important differences in IPF Respir Med 2010 8 296 304 10.1016/j.rmed.2009.09.006 19815403 PMC2856332

[B42] Vielhaber S Brejova A Debska-Vielhaber G Kaufmann J Feistner H Schoenfeld MA Awiszus F 24-months results in two adults with Pompe disease on enzyme replacement therapy Clin Neurol Neurosurg 2011 8 350 357 10.1016/j.clineuro.2010.09.016 21477922

[B43] de Vries JM Brugma JD Ozkan L Steegers EA Reuser AJ van Doorn PA van der Ploeg AT First experience with enzyme replacement therapy during pregnancy and lactation in Pompe disease Mol Genet Metab 2011 8 552 555 10.1016/j.ymgme.2011.09.012 21967859

[B44] Furusawa Y Mori-Yoshimura M Yamamoto T Sakamoto C Wakita M Kobayashi Y Fukumoto Y Oya Y Fukuda T Sugie H Hayashi YK Nishino I Nonaka I Murata M Effects of enzyme replacement therapy on five patients with advanced late-onset glycogen storage disease type II: a 2-year follow-up study J Inherit Metab Dis 2012 8 301 310 10.1007/s10545-011-9393-6 21984055

[B45] Korpela MP Paetau A Löfberg MI Timonen MH Lamminen AE Kiuru-Enari SM A novel mutation of the GAA gene in a Finnish late-onset Pompe disease patient: clinical phenotype and follow-up with enzyme replacement therapy Muscle Nerve 2009 8 143 148 10.1002/mus.21291 19472353

[B46] Van der Beek NA Hagemans ML Reuser AJ Hop WC Van der Ploeg AT Van Doorn PA Wokke JH Rate of disease progression during long-term follow-up of patients with late-onset Pompe disease Neuromuscul Disord 2009 8 113 117 10.1016/j.nmd.2008.11.007 19084399

[B47] Escolar DM Hache LP Clemens PR Cnaan A McDonald CM Viswanathan V Kornberg AJ Bertorini TE Nevo Y Lotze T Pestronk A Ryan MM Monasterio E Day JW Zimmerman A Arrieta A Henricson E Mayhew J Florence J Hu F Connolly AM Randomized, blinded trial of weekend vs daily prednisone in Duchenne muscular dystrophy Neurology 2011 8 444 452 10.1212/WNL.0b013e318227b164 21753160 PMC3146308

[B48] Spurney CF Rocha CT Henricson E Florence J Mayhew J Gorni K Pasquali L Pestronk A Martin GR Hu F Nie L Connolly AM Escolar DM Cooperative International Neuromuscular Research Group Investigators CINRG pilot trial of coenzyme Q10 in steroid-treated Duchenne muscular dystrophy Muscle Nerve 2011 8 174 178 10.1002/mus.22047 21698649 PMC3136634

[B49] Mayhew JE Florence JM Mayhew TP Henricson EK Leshner RT McCarter RJ Escolar DM Reliable surrogate outcome measures in multicenter clinical trials of Duchenne muscular dystrophy Muscle Nerve 2007 8 36 42 10.1002/mus.20654 16969838

[B50] Escolar DM Buyse G Henricson E Leshner R Florence J Mayhew J Tesi-Rocha C Gorni K Pasquali L Patel KM McCarter R Huang J Mayhew T Bertorini T Carlo J Connolly AM Clemens PR Goemans N Iannaccone ST Igarashi M Nevo Y Pestronk A Subramony SH Vedanarayanan VV Wessel H CINRG Group CINRG randomized controlled trial of creatine and glutamine in Duchenne muscular dystrophy Ann Neurol 2005 8 151 155 10.1002/ana.20523 15984021

[B51] Beenakker EA Fock JM Van Tol MJ Maurits NM Koopman HM Brouwer OF Van der Hoeven JH Intermittent prednisone therapy in Duchenne muscular dystrophy: a randomized controlled trial Arch Neurol 2005 8 128 132 10.1001/archneur.62.1.128 15642859

[B52] Scott E Eagle M Mayhew A Freeman J Main M Sheehan J Manzur A Muntoni F North Star Clinical Network for Paediatric Neuromuscular Disease Development of a functional assessment scale for ambulatory boys with Duchenne muscular dystrophy Physiother Res Int 2012 8 101 109 10.1002/pri.520 21954141

[B53] Mayhew A Cano S Scott E Eagle M Bushby K Muntoni F North Star Clinical Network for Paediatric Neuromuscular Disease Moving towards meaningful measurement: Rasch analysis of the North Star Ambulatory Assessment in Duchenne muscular dystrophy Dev Med Child Neurol 2011 8 535 542 10.1111/j.1469-8749.2011.03939.x 21410696

[B54] Mazzone E Martinelli D Berardinelli A Messina S D’Amico A Vasco G Main M Doglio L Politano L Cavallaro F Frosini S Bello L Carlesi A Bonetti AM Zucchini E De Sanctis R Scutifero M Bianco F Rossi F Motta MC Sacco A Donati MA Mongini T Pini A Battini R Pegoraro E Pane M Pasquini E Bruno C Vita G de Waure C Bertini E Mercuri E North Star Ambulatory Assessment, 6-minute walk test and timed items in ambulant boys with Duchenne muscular dystrophy Neuromuscul Disord 2010 8 712 716 10.1016/j.nmd.2010.06.014 20634072

[B55] Mayhew AG Eagle M Scott E Bushby KM Adnan M Muntoni F Sano SJ Trial readiness: clinical interpretability of change scores of the North Star Ambulatory Assessment in Duchenne muscular dystrophy [abstract] Neuromuscul Disord 2012 8 876 877

[B56] Vuillerot C Payan C Girardot F Fermanian J Iwaz J Bérard C Ecochard R MFM Study Group Responsiveness of the motor function measure in neuromuscular diseases Arch Phys Med Rehabil 2012 8 2251 2256 10.1016/j.apmr.2012.05.025 22705238

[B57] Cooperative International Neuromuscular Research Group (CINRG) CQMS advantage [ http://www.cinrgresearch.org/cinrgnetwork/cqms.cfm]

[B58] Grootenhuis MA de Boone J van der Kooi AJ Living with muscular dystrophy: health related quality of life consequences for children and adults Health Qual Life Outcomes 2007 8 31 10.1186/1477-7525-5-31 17553127 PMC1894786

[B59] Vandervelde L Van den Bergh PY Renders A Goemans N Thonnard JL Relationships between motor impairments and activity limitations in patients with neuromuscular disorders J Neurol Neurosurg Psychiatry 2009 8 326 332 10.1136/jnnp.2008.150060 18948363

[B60] Studenski S Perera S Patel K Rosano C Faulkner K Inzitari M Brach J Chandler J Cawthon P Connor EB Nevitt M Visser M Kritchevsky S Badinelli S Harris T Newman AB Cauley J Ferrucci L Guralnik J Gait speed and survival in older adults JAMA 2011 8 50 58 10.1001/jama.2010.1923 21205966 PMC3080184

[B61] Hardy SE Perera S Roumani YF Chandler JM Studenski SA Improvement in usual gait speed predicts better survival in older adults J Am Geriatr Soc 2007 8 1727 1734 10.1111/j.1532-5415.2007.01413.x 17916121

[B62] Inam S Vucic S Brodaty NE Zoing MC Kiernan MC The 10-metre gait speed as a functional biomarker in amyotrophic lateral sclerosis Amyotroph Lateral Scler 2010 8 558 561 10.3109/17482961003792958 20515425

[B63] Claerbout M Gebara B Ilsbroukx S Verschueren S Peers K Van Asch P Feys P Effects of 3 weeks’ whole body vibration training on muscle strength and functional mobility in hospitalized persons with multiple sclerosis Mult Scler 2012 8 498 505 10.1177/1352458511423267 22084490

[B64] Schimpl M Moore C Lederer C Neuhaus A Sambrook J Danesh J Ouwehand W Daumer M Association between walking speed and age in healthy, free-living individuals using mobile accelerometry–a cross-sectional study PLoS One 2011 8 e23299 10.1371/journal.pone.0023299 21853107 PMC3154324

[B65] Motl RW Weikert M Suh Y Sosnoff JJ Pula J Soaz C Schimpl M Lederer C Daumer M Accuracy of the actibelt(^®^) accelerometer for measuring walking speed in a controlled environment among persons with multiple sclerosis Gait Posture 2012 8 192 196 10.1016/j.gaitpost.2011.09.005 21945386

[B66] Wood M Cleary MA Alderson L Velllodi A Changes in gait pattern as assessed by the GAITRite™ walkway system in MPS II patients undergoing enzyme replacement therapy J Inherit Metab Dis 2009 8 Suppl 1 S127 S135 19319660 10.1007/s10545-009-1103-2

[B67] Pickett KA Duncan RP Hoekel J Marshall B Hershey T Earhart GM Washington University Wolfram Study Group Early presentation of gait impairment in Wolfram Syndrome Orphanet J Rare Dis 2012 8 92 10.1186/1750-1172-7-92 23217193 PMC3551701

[B68] Sosnoff JJ Weikert M Dlugonski D Smith DC Motl RW Quantifying gait impairment in multiple sclerosis using GAITRite technology Gait Posture 2011 8 145 147 10.1016/j.gaitpost.2011.03.020 21531562

[B69] Devy R Lehert P Varlan E Genty M Edan G A short and validated multiple sclerosis-specific health-related quality of life measurement for routine medical practice Eur J Neurol 2013 8 935 941 10.1111/ene.12107 23425442

[B70] Cedarbaum JM Stambler N Malta E Fuller C Hilt D Thurmond B Nakanishi A The ALSFRS-R: a revised ALS functional rating scale that incorporates assessments of respiratory function. BDNF ALS study group (phase III) J Neurol Sci 1999 8 13 21 10.1016/S0022-510X(99)00210-5 10540002

[B71] Nève V Cuisset JM Edmé JL Carpentier A Howsam M Leclerc O Matran R SNIP interest in the longitudinal assessment of young Duchenne muscular dystrophy children Eur Respir J 2012 doi:10.1183/09031936.00127712 10.1183/09031936.00127712 23258781

[B72] Hobson-Webb LD Jones HN Kishnani PS Oropharyngeal dysphagia may occur in late-onset Pompe disease, implicating bulbar muscle involvement Neuromuscul Disord 2013 8 319 323 10.1016/j.nmd.2012.12.003 23332114

[B73] Prigent H Orlikowski D Laforèt P Letilly N Falaize L Pellegrini N Annane D Raphael JC Lofaso F Supine volume drop and diaphragmatic function in adults with Pompe disease [letter] Eur Respir J 2012 8 1545 1546 10.1183/09031936.00169011 22654013

[B74] Vianello A Semplicini C Paladini L Concas A Ravaglia S Servidei S Toscano A Mongini T Angelini C Pegoraro E Enzyme replacement therapy improves respiratory outcomes in patients with late-onset type II glycogenosis and high ventilator dependency Lung 2013 8 537 544 10.1007/s00408-013-9489-x 23839583

[B75] Angelini C Semplicini C Ravaglia S Moggio M Comi GP Musumeci O Pegoraro E Tonin P Filosto M Servidei S Morandi L Crescimanno G Marrosu G Siciliano G Mongini T Toscano A the Italian Group on GSDII New motor outcome function measures in evaluation of late-onset Pompe disease before and after enzyme replacement therapy Muscle Nerve 2012 8 831 834 10.1002/mus.23340 22581536

[B76] van der Beek NA Hagemans ML van der Ploeg AT van Doorn PA Merkies IS The Rasch-built Pompe-specific Activity (R-PAct) scale Neuromuscul Disord 2013 8 256 264 10.1016/j.nmd.2012.10.024 23273871

